# Interplay among Oxidative Stress, Methylglyoxal Pathway and S-Glutathionylation

**DOI:** 10.3390/antiox10010019

**Published:** 2020-12-28

**Authors:** Lidia de Bari, Andrea Scirè, Cristina Minnelli, Laura Cianfruglia, Miklos Peter Kalapos, Tatiana Armeni

**Affiliations:** 1 Institute of Biomembranes, Bioenergetics and Molecular Biotechnologies (IBIOM), National Research Council (CNR), 70126 Bari, Italy; 2 Department of Life Environmental Sciencesand, Università Politecnica delle Marche, 60100 Ancona, Italy; a.a.scire@univpm.it (A.S.); c.minnelli@staff.univpm.it (C.M.); 3 Department of Clinical Sciences, Università Politecnica delle Marche, 60100 Ancona, Italy; l.cianfruglia@univpm.it; 4 Theoretical Biology Research Group, Dámvad utca 18, H-1029 Budapest, Hungary; mpkalapos@freemail.hu

**Keywords:** glyoxalase system, glutathione, glutathionylation, mitochondria, methylglyoxal, S-D-lactoylglutathione, redox signaling

## Abstract

Reactive oxygen species (ROS) are produced constantly inside the cells as a consequence of nutrient catabolism. The balance between ROS production and elimination allows to maintain cell redox homeostasis and biological functions, avoiding the occurrence of oxidative distress causing irreversible oxidative damages. A fundamental player in this fine balance is reduced glutathione (GSH), required for the scavenging of ROS as well as of the reactive 2-oxoaldehydes methylglyoxal (MGO). MGO is a cytotoxic compound formed constitutively as byproduct of nutrient catabolism, and in particular of glycolysis, detoxified in a GSH-dependent manner by the glyoxalase pathway consisting in glyoxalase I and glyoxalase II reactions. A physiological increase in ROS production (oxidative eustress, OxeS) is promptly signaled by the decrease of cellular GSH/GSSG ratio which can induce the reversible S-glutathionylation of key proteins aimed at restoring the redox balance. An increase in MGO level also occurs under oxidative stress (OxS) conditions probably due to several events among which the decrease in GSH level and/or the bottleneck of glycolysis caused by the reversible S-glutathionylation and inhibition of glyceraldehyde-3-phosphate dehydrogenase. In the present review, it is shown how MGO can play a role as a stress signaling molecule in response to OxeS, contributing to the coordination of cell metabolism with gene expression by the glycation of specific proteins. Moreover, it is highlighted how the products of MGO metabolism, S-D-lactoylglutathione (SLG) and D-lactate, which can be taken up and metabolized by mitochondria, could play important roles in cell response to OxS, contributing to cytosol-mitochondria crosstalk, cytosolic and mitochondrial GSH pools, energy production, and the restoration of the GSH/GSSG ratio. The role for SLG and glyoxalase II in the regulation of protein function through S-glutathionylation under OxS conditions is also discussed. Overall, the data reported here stress the need for further studies aimed at understanding what role the evolutionary-conserved MGO formation and metabolism can play in cell signaling and response to OxS conditions, the aberration of which may importantly contribute to the pathogenesis of diseases associated to elevated OxS.

## 1. Introduction

To maintain its function and structure, a cell requires energy. In cellular metabolism, energy-producing reactions from biomolecules are controlled by oxido-reductases, a group of enzymes that transfer electrons from donors to electron acceptors. High-energy, reduced molecules deriving from nutrient catabolism are oxidized in the mitochondrial electron transport chain during cell respiration, where oxygen is the final electron acceptor [1]. The reduction of oxygen can occur in two-electron and one-electron forms of reactions, leading to water and reactive oxygen species (ROS) formation, respectively. Since mitochondria are those subcellular compartments where oxygen through terminal oxidation accepts electrons, these are the organelles where the majority of ROS formation takes place. Under physiological conditions, less than 10% of the total oxygen consumption by mitochondria is considered to be emitted as ROS [2,3]. Physiological ROS production, a condition known as “oxidative eustress” (OxeS), occurs in normal cell metabolism and activates redox signaling to induce cell metabolic changes differently from excessive ROS load, namely “oxidative distress” (OxdS) that on the contrary, causes oxidative damage [4]. As free radicals, in general, and ROS in particular, are highly toxic, it is essential for a cell to develop appropriate protective systems that ensure a correct cellular redox balance. However, under pathological conditions, the balance is impaired, thus resulting in a disturbance of cell redox state that can affect the structure and function of cell components. Under conditions of oxidative stress (OxS), protection of cysteine residues against irreversible oxidation is fundamental to block further damage on protein structure and function [5]. The mechanism of protection is S-glutathionylation, a reversible S-thiolation reaction leading to the formation of a mixed disulfide between protein cysteine residue and glutathione. S-glutathionylation can also regulate protein function, this being involved in the stimulation of cell response to OxS [6]. Changes in the relative amounts of reduced glutathione (GSH) and oxidized glutathione (GSSG), the GSH/GSSG ratio, are of primary importance for the induction of protein S-glutathionylation [7].

**Figure 1:**
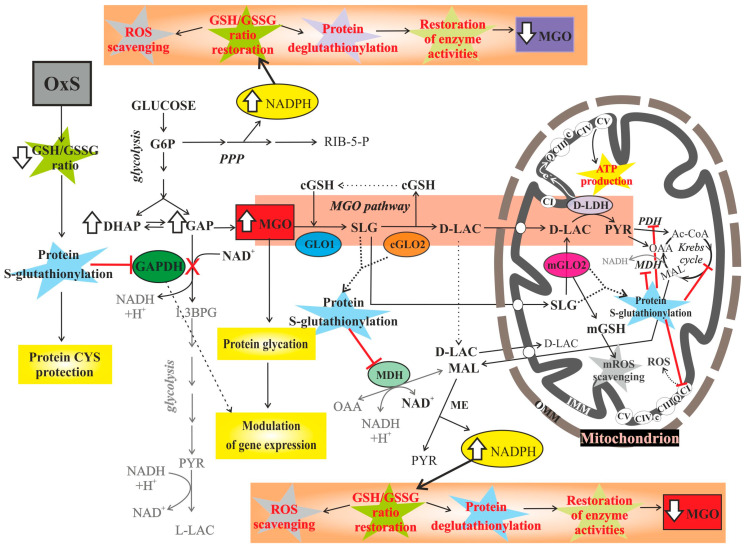
The role of methylglyoxal and methylglyoxal pathway in cell response to oxidative stress. The possible role of MGO and MGO pathway in cell response to OxS are schematically represented. OxS is shown to cause the elevation of MGO level as a result of OxS-induced S-glutathionylation and inhibition of GAPDH. In the presence of non-limiting amounts of cGSH, MGO can feed the MGO pathway, leading to increased SLG and D-LAC formation which can both facilitate the res-toration of the normal redox state. The possible role of MGO as a signaling molecule, through protein modification and gene expression modulation, is also represented. SLG can feed the mGSH pool via mGLO2 reaction, thus contributing to mitochondrial reactive oxygen species (mROS) scavenging. D-LAC oxidation, catalyzed by the mitochondrial flavoprotein D-lactate de-hydrogenase (D-LDH), can sustain mitochondrial ATP production, bypassing the ROS-producing complex I (CI) of the respiratory chain. D-LAC can also cause the efflux of malate (MAL) from mitochondria, thus contributing to NADPH synthesis and ROS detoxification in the cytosol. The role of OxS-induced S-glutathionylation, to which GLO2 and SLG can contribute, in the inhibition of certain cytosolic and mitochondrial enzymes, and protection of protein cysteine residues (CYS) from irreversible deactivation, is also highlighted. The white-filled arrows indicate the enhance-ment/decrease of metabolite levels caused by, or in response to OxS. Grey writings and arrows in-dicate decreased metabolite levels/reactions due to OxS-activated S-glutathionylations. Abbrevia-tions not included in the text: Ac-CoA, acetyl CoA; 1,3BPG, 1,3-bisphosphoglycerate; CIII, coen-zyme Q—cytochrome c reductase (complex III); CIV, cytochrome c oxidase (complex IV); CV, ATP synthase (complex V); Q, coenzyme Q; c, cytochrome c; CYS, cysteine residues; G6P, glucose-6-phosphate; ME, malic enzyme; OAA, oxaloacetate; PYR, pyruvate; RIB-5-P, ribose-5-phosphate.

GSH is a thiol buffer of the cell, utilized both as an antioxidant and a cofactor by many antioxidant enzymes [8]. In the glyoxalase system, a GSH molecule is spent for the detoxification of methylglyoxal (MGO). MGO is a reactive product of carbohydrate, protein and lipid metabolism formed in all cells both under normal and pathological conditions [9]. There are several enzyme-catalyzed or non-enzymatic reactions leading to MGO production [10]. On the contrary, the main way of MGO detoxification is the MGO pathway in which the ubiquitous glyoxalase system, comprising glyoxalase I (GLO1) and glyoxalase II (GLO2), converts MGO into the safe final product D-lactate (D-LAC), a reduced substrate that can fuel mitochondrial respiration [11]. Since D-LAC formation occurs solely by the action of glyoxalase enzymes, its metabolism may be considered as the ultimate step of MGO detoxification. S-D-lactoylglutathione (SLG) is the intermediate of this conversion, being the product of GLO1 and the substrate of GLO2 catalyzed reactions. In the detoxification of MGO, the glyoxalase system uses GSH as a cofactor. MGO at high levels, as occurring in hyperglycemia and other conditions, can react with proteins, DNA and other biomolecules, and therefore is the major precursor of advanced glycation end products (AGEs) which increase OxS [12]. There are several observations supporting the possible involvement of the MGO pathway in redox signaling under OxeS conditions [11,13,14]. In fact, it seems that GLO2 is able to catalyze protein S-glutathionylation using SLG as a substrate [15,16], indicating that a role can be assigned to the glyoxalase system in the protection and/or regulation of protein function in response to OxS. Lastly, the existence of the mitochondrial GLO2 (mGLO2) and the occurrence of D-LAC oxidation by the mitochondrial flavoenzyme D-lactate dehydrogenase (D-LDH) [17] suggest other, but still unexplored, scenarios in cell metabolism and response to OxS in which MGO network could be involved [11] (Figure 1).

Here we describe the relationship between OxS and MGO formation and metabolism, focusing on the mechanism of S-glutathionylation as an important protective and signaling mechanism. The compartment-dependent importance of MGO pathway in relation to oxidative pressure and adaptive metabolic changes has been emphasized, together with the possible role of D-LAC. Additionally, the occurrence of S-glutathionylation in the intramitochondrial space and its relation to pathological events has been addressed. Finally, a strategic role for SLG in cell survival in the course of redox imbalance concomitantly with a positive signaling role for MGO in the reestablishment of cell redox balance in OxeS conditions is proposed.

## 2. Methylglyoxal Pathway and Oxidative Stress

MGO is a physiological, though reactive, alpha-oxoaldehyde, mainly produced as a byproduct of glycolysis at the level of triose-phosphates DHAP (dihydroxy-acetonephosphate) and GAP (D-glyceraldehyde 3-phosphate) [9,18]. Being linked to cell metabolism, the rate of MGO formation depends on the organism, tissue, cell metabolism, and physiological conditions and generally increases under conditions of high glycolysis [19] and hyperglycemia [20]. The dicarbonyl group within MGO easily reacts with the amines of proteins and nucleic acids, forming AGEs and leading to the reduction or loss of molecule function. The accumulation of MGO, referred to as “carbonyl stress” [21], is often associated to several pathological conditions and aging [11,22]. However, MGO, which is constitutively produced, has been suggested to fulfill a physiological role, since certain chaperone proteins require MGO-mediated glycation for becoming active. Thus, it could be advantageous for the cell if a given portion of MGO escapes catabolism and glycates specific proteins [23].

In mammalian cells, the primary route for MGO removal is the glyoxalase system. Indeed, the substrate of GLO1 is the hemithioacetal (MGO-GSH) formed in a spontaneous reaction between MGO and GSH. As the “in situ” activity of GLO1 seems to be directly related to cellular GSH level, it has been suggested that GLO1 might already be complexed with GSH in the catalytic site when it reacts with MGO [23]. Interestingly, in spontaneously hypertensive rats, a delayed decrease of GSH was observed to occur after increased MGO formation, indicating that the primary reason for elevated MGO level could be its increased production, rather than its lower removal due to limited GSH availability [24].

GSH plays a key role in the regulation of cell proliferation and death, the detoxification of xenobiotics and their metabolites, and the modulation of cellular redox signaling through protein S-glutathionylation. The reduction of GSSG to GSH is catalyzed by glutathione reductase (GR), which uses NADPH as an electron donor (GSSG + NADPH + H^+^ ⇄ 2 GSH + NADP^+^). Therefore, NADPH and GSH are essential for maintaining cellular redox homeostasis and biological functions. However, an excessive level of both NADPH and GSH is also implicated in certain pathologies due to the induction of a condition of ‘reductive stress’, which is as harmful as OxS [25].

Under OxdS conditions, which can lead to significant GSH decrease, GLO1 activity might decrease causing MGO concentration increase and cytotoxic effects mainly due to the glycation of proteins and DNA [26]. However, another factor that could limit MGO elimination is the accumulation of GLO1 reaction product, namely SLG, due to reduced GLO2 activity, the rate-limiting enzyme of the glyoxalase system. Finally, cell ability to discard D-LAC by oxidizing it inside mitochondria [17] or exporting it to the extracellular phase is also important to avoid MGO accumulation. In plants, following D-LDH silencing, MGO increase, cytosolic GSH level decrease and growth inhibition have been observed [27,28]. Similarly, to what was found in plants, mammalian D-LDH activity could be equally important for the complete detoxification of MGO. Indeed, both yeast and mammalian mitochondria can take up D-LAC, oxidize it in an energy competent manner by D-LDH and export newly synthesized metabolites from the matrix, among which is malate, in exchange with cytosolic D-LAC through a putative D-lactate/malate antiporter [11,29]. In the cytosol, malate could be converted into pyruvate by the cytosolic malic enzyme, a reaction that, together with the pentose phosphate pathway (PPP), represents the principal source of NADPH, which is essential for the maintenance of redox balance and biosynthesis processes [30]. Thus, D-LAC transport and metabolism inside mitochondria might favor MGO elimination and sustain NADPH production in the cytosol, both events having important implications for the control of GSH/GSSG ratio and cell redox state [31] (Figure 1).

Multifactorial pathologic states, such as neurodegenerative diseases, are commonly characterized by elevated OxS and high levels of AGEs formed mainly by the nonenzymatic glycation of proteins, lipids, or nucleic acids by MGO [23,32], and refs therein]. Even if the formation of AGEs occurs as a part of normal metabolism, excessively high levels of AGEs, including dietary AGEs, have pathologic effects due to their ability to promote OxS and inflammation by binding with cell surface receptors or cross-linking with body proteins, altering their structure and function [33,34]. An elevation in MGO level due to pathologic metabolic imbalance, can then cause both increased AGEs formation [35], GSH level decrease, and ROS generation [12]. Being a reactive electrophile that attacks the functional groups of several macromolecules, MGO can cause lost or decrease in enzyme activity and mutations of DNA and nucleic acids, this resulting in several aberrant processes among which are telomere shortening, loss of heterochromatin, altered gene expression, glycolysis impairment, mitochondrial dysfunction, and cell death via necrosis or apoptosis [36]. Actually, in neurons and astroglia the GLO1 expression and activity was found to decrease in subjects over 50 years old, and decreased GLO1 levels correlate with AGEs accumulation [37]. Thus, increased oxidative stress and inflammation and metabolic impairment such as those occurring in neurodegenerative diseases, as well as in aging, can be the consequence of abnormal MGO formation and metabolism, (see references in [11,32]).

## 3. S-Glutathionylation and Oxidative Stress

### 3.1. The Role of Mitochondrial ROS and Protein S-Glutathionylation in Cell Signaling and Metabolic Adaptation

Over a certain threshold, ROS overwhelm the antioxidant defenses, resulting in OxdS and in the damage of mitochondrial macromolecules, targeting proteins and enzymes, membrane lipids (particularly cardiolipin), and nucleic acids (RNA and DNA) [38]. However, physiologic ROS production is important for ROS-mediated signaling pathways.

**Figure 2:**
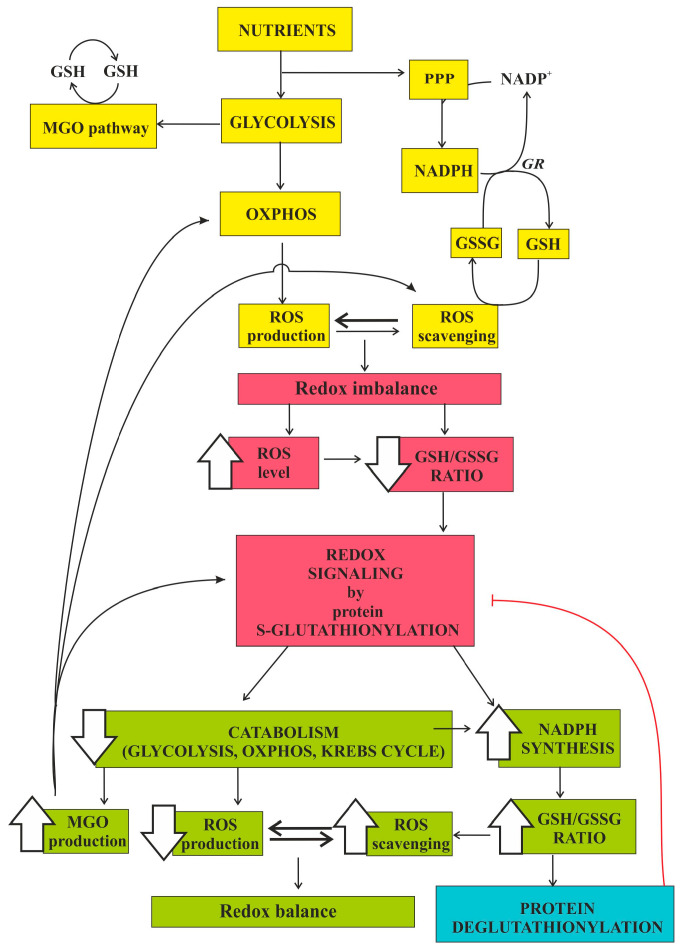
Nutrients can regulate cell metabolism through protein S-glutathionylation-mediated redox signaling. The interconnection between nutrient metabolism, oxidative stress/redox imbalance, and methylglyoxal (MGO) formation and metabolism is schematically represented. Yellow boxes indicate normal nutrient catabolism, which induces ROS production balanced by ROS scavenging. Red boxes indicate a condition of redox imbalance that induces redox signaling through protein S-glutathionylation. Green boxes indicate the metabolic changes that underly cell response to oxidative stress, leading to the restoration of both the redox balance and normal nutrient metabolism via protein deglutathionylation (light blue box). The increase of the MGO level occurring under oxidative stress conditions and its involvement in redox signaling, ROS scavenging, and ATP production via OXPHOS (see Figure 1) are also schematically indicated.

Mitochondria are crucial sites of ROS production, especially H_2_O_2_ and O_2_^−^ [39,40], mainly associated to the activity of the mitochondrial respiratory chain. Respiratory chain activity-linked ROS formation takes place at a higher rate under non-phosphorylating conditions (low ADP-high ATP levels, high electrochemical proton gradient, low oxygen consumption rate), as compared to phosphorylating conditions [38]. H_2_O_2_ is considered a major player in cell redox signaling [41] due to its low reactivity and high capacity to diffuse away from the place of production [42]. Besides catalase, under normal conditions, H_2_O_2_ is efficiently quenched by two main redox systems, glutathione peroxidase (GPX) and peroxiredoxin (PRX), both driven by NADPH, which is produced both in the cytosol and inside mitochondria as a consequence of nutrient metabolism (Figure 2). Thus, H_2_O_2_ links nutrient metabolism to redox signaling. It is now widely accepted that the ability of H_2_O_2_ to modulate mitochondrial activity is mostly due to reversible protein S-glutathionylation [43]. Indeed, the decrease in GSH/GSSG ratio induced by H_2_O_2_ level increase is sensed by glutathione-S-transferases (GSTs) and glutaredoxins (GRXs), which in turn catalyze S-glutathionylation of specific enzymes of glycolysis, mitochondrial respiratory chain, Krebs cycle, fatty acid and amino acid oxidation, directly or indirectly involved in ROS production [41]. H_2_O_2_ can also induce protein S-glutathionylation directly by oxidizing the protein cysteine thiolate anion, which can then be the target for S-glutathionylations (refs in: [41]). Therefore, H_2_O_2_ can regulate cell metabolism through S-glutathionylation.

Key mitochondrial enzymes, including Krebs cycle enzymes, respiratory chain complexes, solute anion and protein import carriers, antioxidant enzymes, and proteins involved in controlling mitochondrial fission and fusion, as well as in apoptosis (see [44] and refs therein) can be regulated by S-glutathionylation. This implies that nutrient metabolism can actively regulate mitochondrial function through ROS-dependent S-glutathionylation (Figure 2). Notably, the activity of complex I of the respiratory chain can be reversibly inhibited by S-glutathionylation, whereas complex II is activated by this modification. Since complex I is one of the most important sites of ROS production, its temporary inhibition by S-glutathionylation lowers OxS and protects mitochondrial structure and function from irreversible damage [44], whereas the contemporary activation of complex II could sustain ATP production under these conditions. Many other mitochondrial enzymes have been identified as targets of S-glutathionylation, among which key enzymes of the Krebs cycle and of fatty acid oxidation. The most important enzyme complexes in the Krebs cycle that respond to redox changes occurring in the matrix are pyruvate dehydrogenase (PDH) and α-ketoglutarate dehydrogenase (KGDH), representing entry points for carbohydrate and amino acid derivatives into the Krebs cycle, respectively. Besides their regulation by phosphorylation and allosteric factors, both the enzyme complexes can also be regulated through S-glutathionylation and deglutathionylation in response to redox changes. S-glutathionylation of PDH and KGDH leads to a reduction in their activity, thus limiting the production of NADH+H^+^ and the entry of electrons into a compromised, ROS-overproducing electron transport chain, thereby preventing the worsening of OxS condition [43]. S-glutathionylation reaction has also been proposed to exert protection of key mitochondrial enzymes from irreversible deactivation by ROS when the pro-oxidant production is short in duration and low in magnitude [43,44].

### 3.2. S-Glutathionylation and Methylglyoxal Synergysm

The sensitivity of S-glutathionylation reactions to the fluctuations of glutathione pool redox state is fundamental for the modulation of cell response and adaptation to OxS [45,46] (Figure 2). Importantly, several glycolytic enzymes are inhibited by S-glutathionylation, among which glyceraldehyde-3-phosphate dehydrogenase (GAPDH), aldolase, phosphoglycerate kinase, pyruvate kinase, triose phosphate isomerase (TPI), and L-lactate dehydrogenase [47,48]. Therefore, protein S-glutathionylation likely slows down glucose catabolism in response to OxS [44]. Importantly, H_2_O_2_ raise can induce the reversible inhibition of GAPDH by S-glutathionylation, slowing down the glycolytic flux and favoring the diversion of glucose-6-phosphate towards branching pathways of glycolysis as the pentose phosphate pathway, important for NADPH production and the restoration of GSH levels. As a result, GSH/GSSG ratio increases, leading to both the decrease in H_2_O_2_ level and the activation of the deglutathionylase activity of glutaredoxins (GRXs) that restores normal metabolic fluxes [41]. However, GAPDH activity is strictly correlated to MGO formation [49] since MGO mainly derives from the non-enzymatic conversion of triose phosphate intermediates of glycolysis [11]. Therefore, OxS through enzymatic protein S-glutathionylation of GAPDH promotes MGO level increase. Consistently with the Szent-Gyorgyi’s theory [50], MGO increase could be functional to the inhibition of cell proliferation when the cell undergoes an OxS condition [51,52], whereas successive GAPDH reactivation via deglutahionylation would remove the inhibition.

Differently from H_2_O_2_ disposal that leads to GSH oxidation into GSSG, MGO detoxification via GLO1 is not associated to GSSG formation, because GSH is “trapped” in the SLG molecule. Since GSSG level increase is required for the induction of protein S-glutathionylation, it can be hypothesized that the sequence of events is as follows: The initial increase in ROS level causes an early GSH oxidation and GSSG formation (GSH/GSSG ratio decrease) followed by the activation of S-glutathionylation of proteins, including GAPDH and MGO level increase. Under these conditions, if sufficient GSH is available, increased MGO-GSH hemithioacetal level could lead to increased formation and accumulation of SLG (and hence of “trapped GSH”) in the cytosol, due to the much higher catalytic activity of GLO1 as compared to GLO2 [53], and/or to the possible inhibition of the cytosolic isoform of GLO2 (cGLO2) under particular conditions such as its temporary binding to negatively charged membrane phospholipids [54]. SLG availability would be relevant for cell response to OxS due to GLO2 ability to catalyze S-glutathionylation of proteins using SLG as substrate [15,16]. Therefore, MGO could have a signaling role, favoring cell response to OxS. The concept of MGO as a signaling molecule is not new. In plants, the activities of glyoxalases and antioxidant defense systems are coordinated in mitigating OxS under abiotic stress conditions, under which both ROS and MGO level increase [55]. Increased MGO either upregulates or downregulates genes involved in signal transduction and in abiotic and biotic stress responses, through the modification of either sensor proteins that induce signal cascades or transcription factors that play a role in biotic and abiotic stress adaptation in plants [56]. Thus, there may be a cross-talk between MGO-responsive and stress-responsive signal transduction pathways [55].

In animals, MGO is emerging to have a powerful ability to change both cell proteome and genome, since the glycation of proteins and the modulation of gene expression are interconnected [57]. Furthermore, it is emerging that certain key metabolic enzymes such as GAPDH [58,59], whose activity can be non-enzymatically inhibited by MGO-mediated modification [60], also have a RNA-regulatory activity correlated to their enzymatic function [58]. In particular, data support the idea that GAPDH is engaged as a regulatory RNA-binding protein when its enzymatic activity is inhibited by MGO in neural precursor cells [61], similarly to what reported in T cells [58]. Thus, a moderate MGO level increase under OxeS conditions could account to the coordination of cell metabolism with gene expression. It has been recently proposed that S-glutathionylation and MGO-mediated glycation, which can both modulate the activity and stability of proteins involved in cellular homeostasis and adaptive stress responses, may act simultaneously and synergistically [45,62]. Both mechanisms have been shown to occur in cellular models and surgical samples of cerebral cavernous malformations, being affected by KRIT1 loss-of-function and influenced by the impairment of cellular redox homeostasis ([45,63] and refs therein), confirming their connection.

For all these reasons, MGO emerges as far more than a biomarker of disease and aging. It can be suggested that, under physiological conditions, a transient increase in MGO level could reflect/signal either OxS conditions, increased rate of glycolysis, or the impairment of key glycolytic enzymes, thus inducing SLG formation, GLO2-mediated S-glutathionylation, and/or D-LAC formation. These together lead to the protection of the active site of key cellular enzymes and favor GSH restoration, thus reestablishing normal redox balance and metabolic fluxes. If the equilibrium between MGO formation and detoxification is lost, significant MGO accumulation can occur, causing GSH depletion and protein glycation with consequent irreversible inactivation of certain enzymes, among which are antioxidant enzymes [64,65], activation of pro-oxidant enzymes [66,67,68], and oxidative damage to key cellular components [11,69]. In other words, as observed in plants, MGO could act as an OxS-induced signaling molecule at low levels, regulating diverse aspects of general metabolism and cellular redox homeostasis, or as a toxic agent at higher concentrations [70].

### 3.3. The Importance of Glyoxalase II

The ability of GLO2 to S-glutathionylate target proteins using SLG as a substrate [15] implies that SLG formation and GLO2 activity play a role in the regulation of cell metabolism and signaling. It is worth noting that MGO pathway is strictly linked to glycolysis and bypasses the second tricarbon part of that. In addition, both glycolysis and glyoxalase system are ubiquitous and evolutionary conserved [23]. Since GLO2 activity is the rate limiting step of the MGO pathway [53], thus limiting the conversion of SLG into D-LAC and GSH recovery, it is feasible that GLO1 plays a role in rapid MGO detoxification, while the regulation of cellular redox state and metabolism relies rather on GLO2 activity. From this point of view, it is not a surprise that the changes in expression and activity of the two glyoxalases rarely match [11,50]. Immature, proliferating tissues, as well as cancer cells [11], display a relatively high GLO1 and low GLO2 activity, whereas differentiated, mature tissues, in which cell anabolism and proliferation are reduced, show lower GLO1 and higher GLO2 activities [71,72]. It is not yet fully explored whether the changes in GLO2 activity depend on the cytosolic or on the mitochondrial isoforms of the enzyme (or both of them). In a study by Scirè et al., it was shown that the cytosolic, but not the mitochondrial, isoform was inhibited by its binding to negatively charged membrane phospholipids [54], this suggesting that the two isoforms can be differently regulated. Notice that two GLO2 isoforms can also be differently expressed in mitochondria and in the cytosol, as found in Arabidopsis thaliana, and show different biochemical characteristics ([50] and refs therein), thus probably playing different roles in cell metabolism. As proposed in [11], if the decrease in total GLO2 activity of highly proliferating tissues reflects a decrease in the activity of the cytosolic isoform only, which is the most abundant isoform in the cell [50], this would determine an increase in the cytosolic level of SLG favoring its import and metabolism by mitochondria, thus feeding the mGSH pool and D-LAC formation in the matrix. This strategy can be helpful for the control of mitochondrial ROS level and the production of energy via oxidative phosphorylation without the involvement of ROS-producing Complex I of the respiratory chain. This would explain why a peculiar GLO2 activity reduction occurs in actively proliferating and cancer cells, giving metabolic, biosynthetic, and antioxidant advantages to the cells. On the other hand, if the increase in total GLO2 activity of differentiated tissues reflects cGLO2 activity increase, this would support the cytosolic (not the mitochondrial) GSH pool and lead to D-LAC formation in the cytosol, followed by its transport and metabolism inside mitochondria, which results in both energy production via oxidative phosphorylation and efflux from mitochondria to the cytosol of malate and other reduced substrates in exchange with cytosolic D-LAC [11].

## 4. Mitochondrial Oxidative Stress Control and the Role of Glyoxalase II Isoform

### 4.1. Mitochondrial Transport of GSH

GSH, alone or with specific enzymes including GR, GPXs, GRXs, GSTs, PRXs, and GLO1 can fulfill several roles in the cells, among others scavenging of ROS and MGO, reduction of protein disulfide bonds, repair of DNA and proteins, and metabolism of metal ions [73,74]. Moreover, GSH can S-glutathionylate certain proteins by binding to specific cysteine residues and generating disulfide bonds, thus regulating protein functions [75]. GSH is synthesized in the cytosol and afterwards distributed in cellular compartments [76]. Mitochondrial GSH represents the 10–15% of total GSH pool [77]. Since mitochondria are one of the most significant sources of ROS, mainly superoxide anion (O_2_^−^) and H_2_O_2_, therefore mGSH is fundamental for the protection of mitochondrial DNA, proteins, and membranes from uncontrolled oxidative processes. In particular, the importance of mGSH relies on its ability to counteract H_2_O_2_, lipid hydroperoxides, or xenobiotics, mainly as a cofactor for GPX or GST. It is not surprising then that a consistent decrease in mGSH level causes mitochondrial dysfunction and OxS [78], events that occur early in several diseases, such as Alzheimer’s disease, and trigger further pathological events [11]. mGSH appears to specifically support cell viability, and its selective reduction was shown to cause ROS production and cell death in both glial and neuronal cells, whereas the depletion of the cytosolic GSH pool did not elicit the same deleterious effects [79]. For the same reason, the impairment of GSH uptake by mitochondria induces cell death in invasive cancer cells [80,81]. This gives the measure of the importance of the MGO pathway, with its cytosolic and mitochondrial enzymes, in the interconnection between cytosolic and mitochondrial metabolite and GSH pools, and hence in cell survival.

Mitochondria lack the enzymes for GSH synthesis and degradation. Since GSH is negatively charged at physiologic pH, it cannot penetrate cellular and mitochondrial membranes. The mitochondrial outer membrane (MOM) possesses both voltage dependent anion channels and translocase of the outer membrane [82]. Cytosolic GSH can pass through both types of channels, allowing an equilibration of the cytosolic GSH pool (cGSH) with the intermembrane space (IMS) GSH pool [83]. Therefore, the IMS GSH pool is likely regulated by cytosolic enzymatic activities of GSH synthesis and degradation/utilization [83,84]. In contrast, mGSH pool is distinct from cGSH and IMS GSH, due to limited transport of glutathione across the IMM, impermeable to most solutes present in the IMS. Unlike MOM, across which a rapid transport of GSH from cytosol to IMS is possible, the rate of glutathione transport across the IMM appears very slow ([83] and refs therein). As demonstrated, the transport of GSH across MOM is not rate limiting for GSH flux across IMM [85], but the identity of the IMM carriers transporting GSH into the matrix against electrochemical gradient is still uncertain. The composition of IMM, which establishes its fluidity, seems to influence the GSH ability to enter mitochondria. In particular, a higher cholesterol/phospholipid molar ratio in the IMM, as detected after prolonged alcohol intake or in certain pathologic states such as Alzheimer’s disease, causes a higher membrane rigidity and a specific decrease in GSH transport and mGSH pool [11,77]. At present, two carriers are implicated in GSH transport, the dicarboxylate carrier (DIC) and the oxoglutarate carrier (OGC), that catalyze the electroneutral exchange of GSH with anions, so that no net transfer of charge occurs across IMM [77]. This has been shown by the reconstitution of recombinant mitochondrial carriers into proteoliposomes and the detection of a reduction of [^3^H]GSH uptake in the presence of DIC and OGC substrates and inhibitors [86]. Since DIC and OGC together were responsible for only 45–50% and 70–80% of the total GSH uptake in liver and in kidney mitochondria, respectively [77,87], the existence of one or more yet unknown mechanisms of glutathione transport in mitochondria is suggested [85]. Moreover, mitochondrial GSH uptake via DIC and OGC has been questioned. Booty and colleagues reported that an excess of GSH did not compete for the transport of the canonical substrates of these carriers, and they were unable to measure direct GSH transport regardless of the acid to basic pH range settings [88]. All in all, the mitochondrial GSH translocator(s) still has/have to be completely identified. So, the role of SLG and mGLO2 in GSH transfer from the cytosol to mitochondria becomes particularly important.

### 4.2. Mitochondrial Transport and Metabolism of S-D-Lactoylglutathone and D-Lactate

Similarly, to the cytosol, the mitochondrial matrix and IMS show high GSH pool. It has been estimated that a GSH:GSSG ratio > 10,000:1, with GSSG present only in trace amounts, occurs in the matrix and IMS, and both compartments can rapidly restore glutathione redox potential after an oxidative challenge, suggesting that GSSG is efficaciously reduced to GSH [83]. Through the sequence of GLO1, GLO2, and D-LDH reactions, MGO is finally converted to pyruvate inside mitochondria (Figure 1). The import of SLG has recently been demonstrated in isolated rat liver mitochondria (RLM) [89], where mitochondrial GLO2 isoforms, found in both the matrix and IMS, can split it into D-LAC and GSH [90,91]. Therefore, the physiological role(s) of GLO2 may be more complex than GLO1. SLG is exclusively synthesized in the cytosol by GLO1 and is membrane impermeable [92,93]. The coordination between the SLG transport activity of IMM carriers and mGLO2 clearly can account for and regulate the transfer of GSH from cytosol into mitochondrion, which could be of primary importance, especially in response to increased mitochondrial ROS production. SLG transport across IMM was first demonstrated in RLM by the use of radiolabeled compounds [89] and shown to differ from GSH transport, suggesting two separate transport systems. The incubation of RLM with SLG caused oxygen consumption, increase in mGSH level, and mitochondrial membrane potential generation, as a result of SLG hydrolysis inside mitochondria, followed by D-LAC formation and oxidation in the matrix by D-LDH [89]. On the other hand, SLG hydrolysis by cGLO2 leads to GSH release and D-LAC formation in the cytosol. D-LAC was shown to enter RLM in symport with a proton, a process catalyzed by a putative D-LAC/H+ symporter [94]. Once in the matrix, D-LAC is oxidized to pyruvate by the flavin enzyme D-LDH that, similarly to succinate dehydrogenase, gives reducing equivalents to complex III of the respiratory chain, through coenzyme Q [94]. Thus, under OxS conditions, the mitochondrial transport and oxidation of D-LAC can decrease mitochondrial transmembrane proton gradient, and at the same time, fuel respiratory chain bypassing complex I. Therefore, the slowing down of glycolysis due to OxS-induced S-glutathionylation of GAPDH, concomitant with the enhancement of the MGO pathway [49] (which bypasses glycolysis), might contribute to mitochondrial ATP generation without inducing high rates of mitochondrial ROS production. D-LAC uptake into mitochondria can be also mediated by two separate mitochondrial antiporters, the putative D-LAC/oxoacid and the D-LAC/malate antiporters, thus causing the efflux of pyruvate or oxaloacetate, and malate, respectively, synthesized in the matrix as a result of D-LAC breakdown [94]. Interestingly, L-lactate (L-LAC) does not induce malate efflux from mitochondria [95], thus probably being a peculiarity of the D-isomer of lactate.

### 4.3. The Possible Role of S-D-Lactoylglutathione and GLO2-Mediated S-Glutathionylation in Cellular Redox Control

SLG has been investigated for many years to ascertain its role in cell metabolism. SLG accumulation, not accompanied by significant changes in the ratio of GLO1/GLO2 activity, occurs during the activation of human platelets [96], and neutrophils [97], when the necessity for GSH and the requirement of energy increase. As described above, this intermediate can accomplish both of these cell requirements. SLG also accumulated in human red blood cells incubated with high concentrations of glucose in vitro [98] and during hyperglycemia associated with diabetes mellitus [99]. SLG can induce some remarkable biological responses, such as the release of histamine from basophils [100], neutrophil movement [101], and stimulus-induced secretion of granules [53]. SLG was shown to regulate microtubule length, due to a non-competitive activation and inhibition of GLO1 and GLO2, respectively, during the activation of neutrophils [97]. Later, it was confirmed that SLG, but not D-LAC or GSH alone or in combination, potentiated in vitro GTP-dependent assembly of microtubules in a cell-free system, in the absence of GLO2 [102]. The involvement of SLG in signal transduction to the cytoskeleton during the functional cell activation has been also proposed [96].

Recently, it was shown that, beside the conversion of SLG into D-LAC, GLO2 might have an additional role in catalyzing S-glutathionylation of target proteins using its natural substrate SLG. Indeed, during the hydrolysis of SLG, D-LAC and the thiolate anion of glutathione (GS^−^) are formed in the active site of GLO2, a process facilitated by the zinc-oxygen interaction [103]. GS^−^ is subsequently protonated by one molecule of water and released as GSH [15]. The formation of GS^−^ from GSH can be a prerequisite for the S-glutationylation of S-OH groups of target proteins, as reported for GST [104], and suggests the following possible mechanisms of GLO2-mediated S-glutathionylation [15]:

Pr−S−OH + GS^−^ → Pr−SSG + OH^−^

Pr−S + GS^−^ → Pr−SSG^−^

The ability of GLO2 plus SLG to S-glutathionylate proteins is of great importance since they can play a still unexplored role in the regulation of protein function both in the cytosol and in mitochondria, with several implications for cell signaling and cytosol-mitochondria crosstalk. The S-glutathionylation of malate dehydrogenase (MDH) and actin by GLO2 in the presence of SLG has been shown in vitro [15]. The catalytic site of GLO2 (both in the presence or absence of GSH) was directly involved in the formation of very stable complexes with these target proteins. Since the cysteine residues of actin and MDH lie very close to GLO2 catalytic site in the presence of GSH, the GLO2-GSH system can be important for promoting S-glutathionylation of these proteins [16]. On the contrary, GLO2 was shown to form a complex with GAPDH with low binding affinity and the involvement of GLO2 active site in this protein-protein association was not observed, suggesting that GLO2-mediated post-translational modification of GAPDH is unlikely.

Since the role of GLO2 in protein S-glutathionylation has not been further investigated, a modest speculation on the possible implications of GLO2-mediated regulation of both cytosolic and mitochondrial protein function may be appropriate. As far as actin is concerned, GLO2 could contribute to the maintenance of the redox turnover of actin (actin-SH/actin-SSG) by promoting reversible S-glutathionylation in response to H_2_O_2_ exposition. It was shown that S-glutathionylation and deglutathionylation maintain actin polymerization/depolymerization dynamics. S-glutathionylation of actin at Cys^374^ prevented globular actin (G-actin) from polymerization while epidermal growth factor-induced deglutathionylation of G-actin increased significantly the rate of its polymerization to form filamentous actin (F-actin) [105,106]. Thus, H_2_O_2_–dependent S-glutathionylation, which can be mediated by GLO2 and other enzymes, promotes actin depolymerization, whereas glutaredoxin 1 activity accounts for its polymerization via deglutathionylation [107]. Actin polymerization and adequate actin fiber assembly promotes membrane translocation and assembly of NADPH oxidase, increasing the activity of the enzyme and so leading to NADPH and O_2_ consumption, and O_2_^−^ and H^+^ release. Actin S-glutathionylation/deglutathionylation balance is then crucial to maintain a healthy level of ROS in the whole body, and is involved in respiratory burst [108] and angiotensin II-induced hypertrophic response [109,110,111]. Hence, the interaction between the glyoxalase system and SLG with cytoskeleton could be an additional way for the regulation of both intracellular and extracellular ROS production.

As mentioned above, GLO2 was also reported to induce the enzymatic S-glutathionylation of MDH in the presence of SLG, a modification that alters both MDH conformation and activity. Interestingly, it was found that the activity of GLO2 decreased in the presence of MDH, probably because the active site of GLO2 gets involved in the S-glutathionylation of MDH and is less available for SLG conversion into D-LAC [15]. In eukaryotic cells, MDH exists in mitochondrial and cytoplasmic isoforms [112]. The importance of MDH relies in its ability to catalyze the NAD/NADH-dependent interconversion of the substrates malate and oxaloacetate, being a key part in both the malate/aspartate shuttle of NADH and in the Krebs cycle within mitochondria. In a recent study, it was found that the S-glutathionylated state of MDH, as well as of several other mitochondrial enzymes and complex I, increases significantly in muscle subjected to exercise, in response to exercise-associated production of oxidants [113]. The transient inhibition of MDH activity by S-glutathionylation is able to reduce NADH+H^+^ transfer from the cytosol into mitochondria by the malate-aspartate shuttle, thus elevating the cytoplasmic redox potential and NADH/NAD^+^ ratio, reducing O_2_ consumption by the respiratory chain and the activity of Krebs cycle [114] (Figure 1). Hence, the S-glutathionylation of MDH, to which GLO2 could contribute, can account for the modification of cell metabolism aimed at the reduction of ROS production by mitochondria.

## 5. Conclusions

The glyoxalase pathway is evolutionarily conserved and involved in the glutathione-dependent detoxification of MGO, a cytotoxic by-product of glycolysis. However, MGO formation and metabolism might play fundamental metabolic and signaling roles (Figure 1). Consistently, the importance of the glyoxalase system and D-LDH in several human pathologies, such as cancer, diabetes, and neurologic diseases is well established [11,115,116,117]. Being MGO formation/elimination mainly dependent on nutrient metabolism and GSH availability, and since nutrient metabolism can actively regulate cell and mitochondrial function through ROS-dependent GSH/GSSG ratio fluctuations that induce protein S-glutathionylation, nutrient metabolism, OxS, and MGO are strictly interconnected and in equilibrium with each other (Figure 2). Then, both increased glycolytic flux ([19] and refs therein), inhibition of key glycolytic enzymes [118], conditions affecting GSH availability and/or glyoxalase system and D-LDH activity, can raise MGO intracellular level. OxdS (overwhelming ROS level) can cause a marked MGO concentration raise which, in turn, can further deplete cGSH pool, react with and inhibit key enzymes among which antioxidant enzymes [119], GLO1 [120], and GAPDH [121], and form AGEs, with dramatic effects leading to cell death [122,123,124]. On the contrary, OxeS (low-level, physiological oxidative stress) implies an adaptive oxidative stress response [4]. Under conditions of OxeS a moderate increase in MGO level could occur, signaling a condition of redox and/or metabolic imbalance. Overall, increased MGO can stimulate cellular metabolic compensatory mechanisms through the glyoxalase system, and/or coordinate cell metabolism with gene expression by MGO-mediated glycation of certain RNA-binding proteins [60,61]. Due to the very high amount of cellular GSH and to the high efficiency of cells to restore GSH from GSSG, MGO level increase probably occurs mainly due to its increased production, rather than its decreased elimination by GLO1 [24]. We posit that MGO level increase could derive from OxeS-induced S-glutathionylation and reversible inhibition of GAPDH [49], aimed at favoring PP pathway for the production of NADPH needed for the restoration of GSH levels. However, the bottleneck of glycolysis generated by the decrease in GAPDH activity slows down glucose utilization, likely causing a deficit in energy production which might be partially compensated, in the presence of an efficient glyoxalase system, by the increase in MGO and ultimately in MGO-derived D-LAC formation, which can be oxidized by mitochondria without the involvement of ROS-producing complex I of the respiratory chain. Under these conditions, the conversion of MGO into SLG would further decrease cGSH level, but could favor GLO2-catalyzed S-glutathionylation of certain proteins, such as MDH and actin, involved in cell redox balance maintenance. Furthermore, MGO formation and metabolism could be functional to the potentiation of mGSH pool through SLG mitochondrial transport and conversion into D-LAC inside mitochondria by mGLO2, thus coping with increased mitochondrial ROS production. Whether cytosolic SLG conversion into D-LAC by cGLO2 occurs, cGSH and mGSH levels would not be affected, but D-LAC transport across IMM in antiport with malate might contribute to NADPH formation in the cytosol, thus supporting cGSH pool restoration. Then, a transient increase in MGO formation and metabolism through the glyoxalase system could signal OxS and be actively involved in cellular antioxidant response and restoration of normal redox conditions. An in-depth knowledge of MGO formation and metabolism is certainly of key importance to formulate novel effective therapeutic strategies for several diseases and for healthy aging. The aim of the present review is to stimulate the study of these still unexplored topics in order to better understand how cells can cope with OxS under normal conditions and identify the aberrant mechanisms leading to pathologic OxS response.

## Abbreviations

Advanced glycation end products (AGEs); cytosolic GSH pool (cGSH); cytosolic glyoxalase II (cGLO2); dihydroxy-acetonephosphate (DHAP); dicarboxylate carrier (DIC); D-lactate (D-LAC); D-lactate dehydrogenase (D-LDH); glyceraldehyde 3-phosphate (GAP); glutathione peroxidase (GPX); glutaredoxins (GRXs); glutathione reductase (GR); glutathione-S-transferase (GST); glyceraldehyde-3-phosphate dehydrogenase (GAPDH); glyoxalase I (GLO1); glyoxalase II (GLO2); hemithioacetal (MGO-GSH); inner mitochondrial membrane (IMM); intermembrane space (IMS); α-ketoglutarate dehydrogenase (KGD); mitochondrial GLO2 (mGLO2); L-lactate (L-LAC); malate dehydrogenase (MDH); methylglyoxal (MGO); mitochondrial GSH pool (mGSH); mitochondrial outer membrane (MOM); oxidized glutathione (GSSG); oxidative eustress (OxeS); oxidative distress (OxdS); oxidative stress (OxS); oxoglutarate carrier (OGC); pentose phosphate pathway (PPP); peroxiredoxin (PRX); pyruvate dehydrogenase (PDH); rat liver mitochondria (RLM); reactive oxygen species (ROS); reduced glutathione (GSH); S-D-lactoylglutathione (SLG); triose phosphate isomerase (TPI).
